# Impact of Editor’s Choice Selections on Review Articles in the “Sensory and Motor Neuroscience” Section of Brain Sciences in 2025

**DOI:** 10.3390/brainsci16101055

**Published:** 2026-09-30

**Authors:** Bernadette Murphy

**Affiliations:** Faculty of Health Sciences, Ontario Tech University, Oshawa, ON L1G 0C5, Canada; bernadette.murphy@ontariotechu.ca

The leading review articles for 2025 are clustered [1] into two groups. The first cluster focuses on clinical conditions. The clinical themes include metabolic dysfunction and inflammation in migraine and fibromyalgia; the etiology of small-fiber neuropathy; the handling of acute vertigo and dizziness in the emergency department; clinical frameworks and network dysfunction related to tremor; magnetic resonance imaging for early diagnosis and monitoring of movement disorders; and the impact of glaucoma on the brain.

“Current Trends in Pediatric Migraine: Clinical Insights and Therapeutic Strategies” (Contribution 1) reviews the pathophysiology of pediatric migraines, including genetic predispositions, neuroinflammation, and gut microbiota dysbiosis. Therapeutic approaches are evaluated, including conventional and emerging pharmacological treatments, nutraceuticals, and non-pharmacological approaches. Evidence-based lifestyle interventions for migraine prevention and management are discussed, including stress management, diet, hydration, sleep, exercise, screen time moderation, and cognitive behavioral therapy. The paper reviews the long-term prognosis and follow-up of pediatric migraine patients. The study discusses the importance of early diagnosis, and tailored multidisciplinary care to prevent chronic progression.

On a related theme in adults, the review “Metabolic Dysfunction and Dietary Interventions in Migraine Management: The Role of Insulin Resistance and Neuroinflammation—A Narrative and Scoping Review” (Contribution 2) reviews migraine pathogenesis and emerging evidence demonstrating associations between migraine, insulin resistance, and hyperinsulinism. Hyperinsulinism results in TRPV1 sensitization, increased CGRP release, oxidative stress, mitochondrial dysfunction, and systemic inflammation which can all contribute to migraine. Metabolic dysfunction (obesity and insulin resistance) can increase the severity and frequency of migraines. Evidence is described for the role of anti-inflammatory, Mediterranean, and ketogenic diets, for reducing migraine frequency by decreasing inflammation and oxidative stress, improving mitochondrial function, and stabilizing glucose metabolism.

“Neuroinflammatory and Immunological Aspects of Fibromyalgia” (Contribution 3) reviews evidence for the role of neuroinflammation and immune changes in fibromyalgia. The interactions between the neural pain networks and the immune system in fibromyalgia appear to be bidirectional and operate both centrally and peripherally. The review also discusses the growing body of work on the dorsal root ganglia and the role of maladaptive microglial cell activation.

“Small-Fiber Neuropathy: An Etiology-Oriented Review” (Contribution 4) reviews the causes of small-fiber neuropathy (SFN), with metabolic (diabetes mellitus/impaired glucose tolerance) and idiopathic causes being the most common. This narrative review provides a general picture of SFN and concludes that while idiopathic causes are the most prevalent form, patients with infectious and autoimmune conditions who have pain with neuropathic features should be investigated for SFN.

“Acute Vertigo, Dizziness and Imbalance in the Emergency Department—Beyond Stroke and Acute Unilateral Vestibulopathy—A Narrative Review” (Contribution 5) is timely. New-onset vertigo, dizziness and gait imbalance represent 2.1–4.4% of all patients presenting to the emergency department, but the multiple causes create diagnostic challenges. The diagnostic criteria for acute vestibular syndrome (AVS, i.e., presenting with acute-onset prolonged vertigo/dizziness with accompanying gait imbalance, motion intolerance, nausea/vomiting, with or without nystagmus) includes a differential diagnosis of vertebrobasilar stroke and acute unilateral vestibulopathy. Non-neurological causes can also cause AVS. These include drug side-effects or intoxication, electrolyte disturbances, cardiac disease, severe anemia, carbon monoxide poisoning, and endocrine disorders. Other neurological diseases, including demyelinating CNS diseases, posterior fossa mass lesions, acute thiamine deficiency and vestibular migraine disorders can also result in AVS or episodic vertigo/dizziness. Additionally, the review points out that acute physiological abnormalities (e.g., hypotension, fever, severe anemia) may unmask existing vestibular impairments that were well-compensated.

“Tremor: Clinical Frameworks, Network Dysfunction and Therapeutics” (Contribution 6) describes how tremor is clinically diverse with overlapping pathophysiologies. This review synthesized recent advances in epidemiology, classification, pathophysiology, and treatment of tremor syndromes. It discusses advances in diagnostic precision due to the use of electrophysiology, neuroimaging, wearable sensors, and artificial intelligence. The causes, syndromes and mechanisms of essential tremor, parkinsonian tremor, dystonic tremor, task-specific tremor, orthostatic tremor, and functional tremor are examined. The limitations of current rating scales and the potential for emerging biomarkers are assessed, as well as the need for pathophysiologically based diagnostic criteria to advance tremor diagnosis and care.

“Advanced Magnetic Resonance Imaging for Early Diagnosis and Monitoring of Movement Disorders” (Contribution 7) evaluates emerging magnetic resonance imaging (MRI) modalities including neuromelanin-sensitive imaging, diffusion-weighted imaging, magnetization transfer imaging, and relaxometry, and their ability to provide biomarkers to detect early microstructural degeneration, iron deposition, and connectivity disruptions in key regions like the substantia nigra. These techniques will enable earlier and more accurate differentiation of movement disorders. These movement disorders include Parkinson’s disease, progressive supranuclear palsy, multiple system atrophy, corticobasal degeneration, Lewy body and frontotemporal dementia, Huntington’s disease, and dystonia. The ability of MRI to provide objective metrics to track disease progression and assess therapeutic efficacy is discussed. The challenges presented by the absence of standardized protocols which limit the integration of these MRI techniques into routine clinical practice are emphasized, and the review advocates for researchers to standardize protocols to enable personalized treatment strategies.

“Beyond the Eye: Glaucoma and the Brain” (Contribution 8) synthesizes emerging evidence that the pathophysiology of glaucoma may extend to trans-synaptic neurodegeneration along the visual pathway and structural changes within central brain regions, including the lateral geniculate nucleus and visual cortex. Glaucoma is traditionally classified as an ocular disease characterized by progressive retinal ganglion cell (RGC) loss and optic nerve damage. This intriguing review uses the phrase ‘brain involvement’ to underscore central changes that accompany or follow retinal ganglion cell loss, and they are not trying to redefine glaucoma as a primary cerebral disorder. Glaucoma patients may exhibit alterations in brain connectivity and cortical thinning, aligning it more closely with neurodegenerative disorders such as Alzheimer’s and Parkinson’s disease. The reviewers suggest that shared mechanisms—such as mitochondrial dysfunction, chronic inflammation, and impaired axonal transport—may link glaucomatous optic neuropathy to central nervous system (CNS) pathology. These could open new therapeutic avenues, such as repurposing neuroprotective and neuroregenerative agents, targeting not only intraocular pressure (IOP) but also broader CNS pathways.

The second cluster of reviews focuses on neurocomputation and executive function. One of the reviews entitled “Neurocomputational Mechanisms of Sense of Agency: Literature Review for Integrating Predictive Coding and Adaptive Control in Human–Machine Interfaces” (Contribution 9) synthesizes computational models—including predictive coding, Bayesian inference, and optimal control theories—to establish a unified neurocomputational framework for the sense of agency (SoA). SOA is the subjective experience of controlling an individual’s own actions and the consequences of those actions. The review details how SoA arises from hierarchical inference processes that continuously compare top-down motor predictions with bottom-up sensorimotor feedback. Disruptions in these internal predictive dynamics are shown to underlie the pathophysiology of motor and neuropsychiatric conditions, such as functional movement disorder. The review also highlights the somato-cognitive action network (SCAN) as a key neural substrate integrating motor intention with cognitive control. Mapping these computational mechanisms can aid the development of adaptive human–machine interfaces, closed-loop biofeedback, and extended reality (XR) rehabilitation paradigms designed to restore agency in clinical populations.

The review entitled “A Standard Operating Procedure for Dual-Task Training to Improve Physical and Cognitive Function in Older Adults: A Scoping Review” (Contribution 10) seeks to address the lack of official clinical guidelines for dual-task (DT) training which involves executing physical exercises while simultaneously performing a secondary motor or cognitive task. DT has demonstrated benefits for both motor and cognitive domains in older adults. This scoping review analyzed 44 studies published over the past decade to establish a standardized protocol for healthy older populations. Methodologically, the authors systematically synthesized key exercise variables including training frequency, intensity, duration, and secondary task selection. The resulting Standard Operating Procedure (SOP) recommends pairing postural balance or resistance exercise with a concurrent mental tracking task, prescribed at two 60 min sessions per week for a minimum of 12 weeks to ensure measurable functional improvements. This standardized framework provides a practical, evidence-based reference to unify future clinical research and guide real-world training implementations.

A related review on “Physical Activity and Its Effects on Executive Functions and Brain Outcomes in Children: A Review” (Contribution 11) synthesizes current evidence on the relationship between physical activity, executive functions, and brain outcomes in children. Acute exercise was found to transiently improves cognition via increased neurotransmission and oxygenation, whereas chronic activity promotes lasting structural and functional brain adaptations. Both neurotypical children and those with ADHD experienced cognitive benefits, with inhibitory control demonstrating the greatest overall responsiveness. The review notes the heterogeneity across current protocols and the need to determine standardized exercise dosing parameters (intensity, duration, type) to optimize physical activity to improve clinical and educational outcomes.

Finally, “Exploring Imagined Movement for Brain–Computer Interface Control: An fNIRS and EEG Review” (Contribution 12) reviews recent advancements in utilizing electroencephalography (EEG) and functional Near-Infrared Spectroscopy (fNIRS) to restore motor function by decoding Motor Imagery (MI) movements for the control of Brain–Computer Interfaces (BCIs). The review highlights that EEG provides high temporal resolution but is sensitive to noise, whereas fNIRS offers greater robustness to electrical and motion artifacts but suffers from an inherent physiological delay between MI and the fNIRs signal. Recent advances in machine learning, particularly deep learning, alongside hybrid EEG–fNIRS systems, have improved the accuracy and feasibility of online MI decoding by combining temporal precision with spatial specificity. While predicting online imagined movement is feasible, it remains less reliable than executing actual movement, underscoring the need for continued refinement in neuroimaging integration and signal classification to accelerate practical neuroprosthetic applications.

These reviews cover diverse topics from recent developments in diagnosis and/or clinical management of neuropathologies (migraine, fibromyalgia, small fiber neuropathy, tremor, acute vertigo and dizziness), new perspectives on existing problems (glaucoma), optimizing physical activity protocols to improve executive function, neurocomputation of the sense of agency and enhancing the ability to decode signals related to motor imagery to improve BCI. These reviews highlight novel developments and perspectives in fundamental areas of neuroscience.
